# Biogenic propane production by a marine *Photobacterium* strain isolated from the Western English Channel

**DOI:** 10.3389/fmicb.2022.1000247

**Published:** 2022-10-25

**Authors:** Felicity Currie, Matthew S. Twigg, Nicholas Huddleson, Keith E. Simons, Roger Marchant, Ibrahim M. Banat

**Affiliations:** ^1^School of Biomedical Sciences, Ulster University, Coleraine, United Kingdom; ^2^SHV Energy, Hoofddorp, Netherlands

**Keywords:** propane, biogas, microbial, marine bacteria, *Photobacterium*

## Abstract

Propane is a major component of liquefied petroleum gas, a major energy source for off-grid communities and industry. The replacement of fossil fuel-derived propane with more sustainably derived propane is of industrial interest. One potential production route is through microbial fermentation. Here we report, for the first time, the isolation of a marine bacterium from sediment capable of natural propane biosynthesis. Propane production, both in mixed microbial cultures generated from marine sediment and in bacterial monocultures was detected and quantified by gas chromatography–flame ionization detection. Using DNA sequencing of multiple reference genes, the bacterium was shown to belong to the genus *Photobacterium*. We postulate that propane biosynthesis is achieved through inorganic carbonate assimilation systems. The discovery of this strain may facilitate synthetic biology routes for industrial scale production of propane *via* microbial fermentation.

## Introduction

Liquefied petroleum gas (LPG) is a mixture of propane and butane, traditionally derived from oil refining or natural gas processing and is an essential off-grid fuel source for rural communities and business worldwide (Raslavičius et al., 2014; Aberilla et al., 2020). As LPG possesses a low carbon to hydrogen ratio it is clean-burning and generates lower amounts of carbon dioxide per capacity of heat produced and as such can provide significant carbon savings in comparison to solid and liquid fuel options such as coal and petroleum (Ristovski et al., 2005; Johnson, 2009). When utilized for transportation, LPG has further advantages over fossil alternatives producing 14 and 10% less CO_2_ than petrol and diesel, respectively, while producing virtually no PM0.25, NOx, or SOx (Ristovski et al., 2005; Saraf et al., 2009). LPG produced from renewable feedstocks such as plant and vegetable waste material is termed BioLPG (Johnson, 2015, 2019). BioLPG is a drop-in product that can be used with the existing distribution infrastructure and without any modification to existing equipment and networks (Johnson, 2019). Currently, commercial production of BioLPG is based on the recovery and processing of bio-propane as a 5% side stream from hydrotreating waste and vegetable oils for renewable diesel production, known as the HVO process (Neste Corporation, 2015; Johnson, 2019). Switching to bioLPG could further decarbonize European energy needs, achieving up to 80% reduction in CO_2_ emissions compared to fossil LPG (Bhardwaj et al., 2013; Prussi et al., 2020). The first quantities of bio-propane came to the United Kingdom in 2018 with an initial agreement between *Neste Oil* and *SHV Energy* to supply and distribute 160,000 tonnes over 4 years (Neste Corporation, 2015). Additional capacity from existing and planned HVO plants is in progress, mainly driven by the growth of Sustainable aviation fuels which uses the same core process technology (Guo and Song, 2019). The available quantity, however, only meets a fraction of the 1.1 million tonne existing United Kingdom LPG market needs.

An alternative route to bioLPG is through synthetic biology, but although researchers have genetically engineered bacteria capable of the production of propane, yield restrictions due to product or substrate toxicity, and maintaining cell integrity at high product concentrations remain a major challenge in industrial biotechnology (Kallio et al., 2014; Menon et al., 2015; Sheppard et al., 2016; Amer et al., 2020; Yunus et al., 2022). To date, no natural biosynthetic pathways to produce propane have been discovered. It is, however, an accepted view in geochemistry that there is evidence for the biological formation of ethane and propane in ocean sediments, this is best justified by microbial production of these gasses (Hinrichs et al., 2006; Amer et al., 2020). These findings imply that multiple substrates and mechanisms may be associated with the formation of hydrocarbons. Microbial production of C2 and C3 hydrocarbons is plausible, and naturally occurring microorganisms might be better adapted to survive at high product concentration. These findings also heavily indicate that a naturally occurring propane-producing bacterium will be found in an anoxic environment and as such any study wishing to identify such organisms should focus on isolation from these environments.

After sampling differing anoxic environments where microbial biogenic propane production could predictably take place, we present, for the first time, clear evidence that naturally occurring microorganisms from marine sediment are capable of propane biosynthesis and identify a specific strain of *Photobacterium* that is carrying out the process. The natural propane biosynthesis identified here was achieved using simple feedstocks, which offers the prospect of the development of a scalable, economic process for propane production through synthetic biology. We also identified several genes that may contribute to potential biochemical pathways for propane production.

## Materials and methods

### Sampling and generation of mixed microbial cultures

With the exception of sample FC.2, terrestrial soil samples were obtained using a soil auger. Sample FC.2 (anaerobic digest) was obtained directly from an agricultural anaerobic digester. Marine sediment sample FC.4 was obtained using a box corer. All samples were immediately placed in cool boxes for transport to the laboratory where they were routinely stored at 4°C. Inoculum slurry was generated within 24 h of obtaining each sample. To form an inoculum slurry approximately 20 g (wet weight) of each terrestrial sample was mixed with 25 ml sterile phosphate-buffered saline (PBS) (*Thermo Scientific*) at pH 7.0. For marine samples, the slurry was generated under the same conditions; however, artificial seawater containing marine salts (*Merck*) at 30 gl^−1^, pH 7.4 was used in place of PBS. Initial liquid microbial cultures were prepared, in triplicate, in 20 ml GC headspace vials. Eight milliliter of either modified R2A or Zobell Marine Medium was inoculated with 2 ml of slurry generated from terrestrial or marine samples, respectively. For medium components see Supplementary Tables S1, S2. Prior to dispensing, the media was boiled to degas and the indicator resazurin was included to monitor anaerobic conditions. Samples were incubated, under micro-aerobic/anaerobic conditions in tightly sealed headspace vials without shaking at 4°C for 11 weeks, before analysis for propane in the headspace above the live cultures. Triplicate vials containing 10 ml of uninoculated media served as negative controls.

Mixed microbial cultures were maintained by subculturing 500 μl of the initial culture in 9.5 ml fresh media within GC headspace vials. Mixed microbial cultures were incubated without shaking at either 4°C for a minimum of 5 weeks or at 20°C for 7 days depending on experimental requirements. For propane analysis, negative controls were prepared as previously described with the addition of 5 mM sodium azide (*Merck*).

### Isolation and routine culture of marine bacterial strains

Solid media was prepared as discussed in section “Sampling and generation of mixed microbial cultures”. with the addition of 1.5% (w/v) agar (*Merck*). After analysis for propane, a 10-fold dilution series of the mixed microbial culture was prepared in R2A or modified Zobell Marine media and 100 μl of 10^−1^, 10^−2^, and 10^−3^ dilutions were spread on the surface of solid media. After being allowed to dry, the agar plates were placed upside down in a sterile anaerobic chamber and a GasPak anaerobe sachet and CO_2_ indicator strip added before the chamber was sealed. Plates were incubated at 4°C for 10 weeks under anaerobic conditions. Individual bacterial species were isolated by repeated colony picking and sub-culture on solid media at 4°C for 10 weeks under anaerobic conditions. For propane analysis, all resultant bacterial strains were cultured in 10 ml liquid medium modified depending on individual experimental conditions for 7 weeks at 4°C. The positive control for propane analysis was a mixed microbial culture and the negative control, a mixed microbial culture treated with NaN_3_.

### Gas chromatographic analysis of microbially produced propane

Propane was analyzed by gas chromatography (GC) on a Varian 450 GC with flame ionization detection (FID). A CombiPAL autoinjector fitted with a gastight syringe was used to sample 250 μl headspace gas above the live bacterial cultures. To confirm the identity of propane, chromatography was carried out on two different column chemistries - a Zebron ZB-624 column (30 m × 0.32 mm × 1.8 μm) (*Phenomenex*) and a GasPro SI PLOT (30 m × 0.32 mm × 1.8 μm) (*Agilent*). GC conditions are provided in Supplementary Table S3. Propane was identified by comparison of retention time with an authentic standard, Messer^®^ CAN*GasPro*pane 99.95% (*Merck*) and quantified using a six-point calibration curve. For the calibration curve, a tenfold dilution series to 10^−3^ was prepared in gas-tight headspace vials fitted with butyl rubber septa, using a gas-tight syringe. Concentrations for the 6-point calibration curve of 40, 30, 20, 15, 10, and 5 ppm were prepared from the 10^−3^ standard concentration in headspace vials containing 10 ml liquid growth medium. The limit of detection for propane, 3 times the standard deviation of multiple data points along the baseline divided by the slope was 0.816 ppm.

### Strain identification

Bacterial isolates were initially identified by the sequencing of the gene encoding the 16S sub-unit of ribosomal RNA (16S rRNA). Chromosomal DNA was extracted from each strain of interest using a DNeasy UltraClean Microbial Kit (*Qiagen*) as per the manufacturer’s instructions. Polymerase Chain Reaction (PCR) amplification and subsequent sequencing of the 16S rRNA gene were carried out *via* the methodology previously described by Twigg et al., (2018) using primers 9bfm and 1512uR (Mühling et al., 2008; Twigg et al., 2018). Phylogenetic identification was based upon BLASTn analysis against the EZBioCloud 16S rRNA database.

### Phylogenetic data analysis

The 16S rRNA sequence of *Photobacterium* sp. FC4.9 along with the 16S rRNA sequences for the other recognized species within the *Photobacterium* genus were aligned using MUSCLE software (Edgar, 2004). This alignment was used to construct a maximum likelihood phylogenetic tree using the Tamura-Nei nucleotide substitution model (Edgar, 2004). Non-uniformity of evolutionary rates among sites was modeled by using a discrete Gamma distribution with 5 rate categories and by assuming that a certain fraction of sites were evolutionarily invariable. Bootstrap analyzes were carried out using 1,000 replications and a bootstrap of ≥75% was used to provide the confidence estimation for clades in the phylogenetic tree.

Reference genes *gyrB*, *rpoD*, *rpoA,* and *recA* were identified from the whole genome sequence of *Photobacterium* sp. FC4.9. Whole genome sequencing was provided by MicrobesNG and carried out as per the company’s methodologies using an *Illumina* sequencing platform. The individual sequences for each reference gene of *Photobacterium* sp. FC4.9 was uploaded to the NCBI nucleotide database. Using the methodology previously described for 16S rRNA, each reference gene was aligned with those from the species that comprise the Profundum clade of the *Photobacterium* genus, and a maximum likelihood phylogenetic tree was generated from the alignment. Molecular Evolutionary Genetic Analysis (MEGA) version 11.0.11 software was used to generate both multiple sequence alignments and phylogenetic trees (Labella et al., 2018; Stecher et al., 2020; Tamura et al., 2021). Phylogenetic trees were subsequently annotated using interactive Tree of Life (iTOL) version 5 software (Letunic and Bork, 2021).

### Statistical analysis

Statistical analysis of propane production by both enrichment cultures and individual isolates was determined by one-way ANOVA followed by Dunnett’s *post hoc* testing. A value of *p* ≤ 0.05 was considered statistically significant. All statistical analysis was carried out with the aid of Prism Version 9.3.1 (350) (GraphPad Software, San Diego, CA, United States).

## Results

### Sampling and mixed microbial culture preparation

Soil or sediment samples were obtained from terrestrial and marine locations where *in situ* conditions were predicted as potential sources for microbial alkane production. Details of all samples are provided in Table 1. After 11 weeks incubation at 4°C microbial growth, either as a biofilm at the medium surface or on the surface of the settled environmental matrix, was observed visually in all the initial cultures except for the Canadian gas field sample. Additionally, no microbial growth was observed in uninoculated controls.

**Table 1:** Samples were collected from 5 sites where environmental conditions were thought to be promising for microbial propane production.

Sample #	Environmental niche	Location	Type	Approx. amount
FC. 1	Peat bog	Aghadowey, Co. Londonderry, United Kingdom (55°1′44”N; 6°38′54”W). Approx. 13 cm depth below soil surface, above clay layer	Soil core sample	2 l soil
FC. 2	Anaerobic digest	Organic Dairy Farm, Aghadowey, Co. Londonderry, United Kingdom (55°1′44”N; 6°38′54”W)	Agricultural digester sample	3 l digest slurry
FC. 3	Gas field sediment	Medicine Hat, Alberta, Canada	Soil core sample	1.5 l sediment
FC. 4	Marine sediment	L4 sample site, Western English Channel (50°15.0’N; 4°13.0’W). Approx. 54 m below sea level.	Box grab sample	2 l sediment
FC. 5	Wetland soil	Rathlin Island, Co. Antrim, United Kingdom (55°17′14”N; 6°15′23”W). Approx. 1 m depth below soil surface.	Soil core sample	4 l soil

### Propane detection within initial mixed microbial cultures

**Figure 1:**
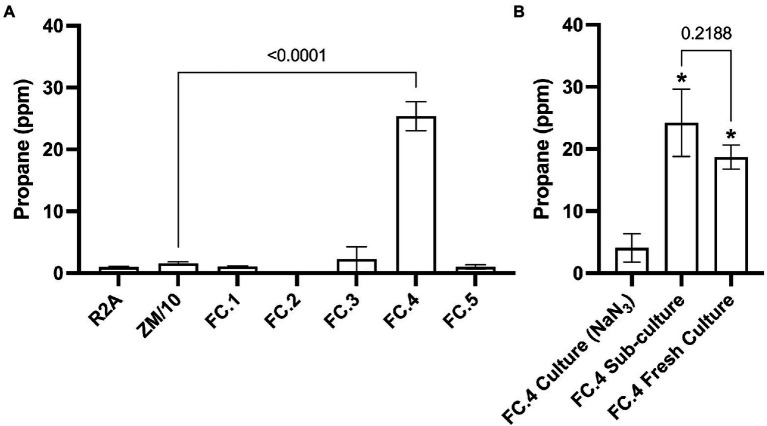
GC-FID detection of propane gas in enrichment cultures generated from environmental samples. **(A)** Significantly increased levels of propane were detected in an cultures generated from a marine sediment sample (FC.4) compared to the uninoculated negative control. **(B)** Significantly increased levels of propane were detected FC.4 cultures compared to the negative control treated with NaN_3_ biocide (One-way ANOVA with Dunnett’s *post hoc* test *p ≤ 0.05, *n* = 3), confirming that propane production was biotic. No significant difference in propane levels were found in a sub-culture from FC.4 or a fresh culture generated using the same marine sediment sample.

Headspace analysis for propane production in the initial mixed microbial cultures generated from each sample location was carried out *via* GC–FID. Propane was identified and quantified by comparison with an authentic propane standard by GC on a Zebron ZB-624 GC column. Propane was detected in initial mixed microbial cultures derived from marine sediment samples (FC.4) at a significantly increased level in comparison to sterile media controls (25.39 ppm ± 2.33, *p* ≤ 0.0001, Figure 1A). No significant propane production was observed in initial mixed microbial cultures generated from the remaining environmental samples (Figure 1A). To confirm the identity of the detected peak was indeed propane, retention time comparison to a propane standard was carried out using two different column chemistries, ZB-624 and GasPro SI PLOT. On both column chemistries, the peak detected in the enrichment culture eluted with the same retention time as the standard propane, at 2.04 and 3.11 min, respectively (Supplementary Figure S1).

There remained the possibility that the observed propane in the marine sediment mixed microbial culture was leaching from the inorganic sediment. To discount this, a sub-culture of the original propane-producing marine sediment culture; a fresh mixed microbial culture generated from an inoculum of the marine sediment sample; and a sub-culture of the original marine sediment culture treated with the biocide sodium azide (NaN_3_) were subjected to GC-FID headspace analysis. Propane was detected in both the sub-cultured and fresh marine sediment mixed microbial cultures at a level significantly higher than that detected in the NaN_3_ treated sample (*p* = 0.0011 and 0.0057, respectively, Figure 1B). No significant difference in propane production was observed between the sub-cultured and fresh marine sediment cultures (*p* = 0.2188, Figure 1B). As propane was consistently not detected in negative control samples inoculated with live cells and then treated with a bactericide (sodium azide) we are confident that propane production was indeed through microbial biosynthesis.

### Isolation and identification of bacterial strains from propane-producing mixed microbial cultures

Twenty strains with unique colony morphologies were isolated from a propane-producing mixed microbial culture following an 11-week incubation at 4°C on solid media supplemented with pre-sterilized marine sediment. Each strain was phylogenetically identified by amplification and subsequent sequencing of the 16S rRNA reference gene. Analysis of sequencing chromatograms from 7 of these strains showed a lack of pure culture. The remaining 13 were deemed to be pure monocultures and a phylogenetic identification of each based upon BLASTn analysis against the EZBioCloud 16S rRNA database can be seen in Table 2. All 16S rRNA gene sequences were uploaded to the NCBI nucleotide database (accession numbers, Table 2).

**Table 2:** Twenty individual bacterial strains were isolate from the marine sediment sample where microbial propane production was identified (FC.4).

Strain #	Accession Num. (NCBI GenBank)	Identification top hit *via* BLASTn (accession num.)	Pairwise similarity (%)	Query coverage (%)
FC4. 1	ON756096	*Halomonas titanicae* BH1 (AOPO01000038)	99.92	94.8
FC4. 2	ON756097	*Halomonas titanicae* BH1 (AOPO01000038)	99.92	94.0
FC4. 3	ON756098	*Halomonas titanicae* BH1 (AOPO01000038)	99.92	92.7
FC4. 4	Sequence analysis revealed mixed culture			
FC4. 5	Sequence analysis revealed mixed culture			
FC4. 6	ON756099	*Halomonas glaciei* DD 39 (AJ431369)	100.00	63.6
FC4. 7	ON756100	*Photobacterium frigidiphilum* SL13 (AY538749)	99.71	93.1
FC4. 8	Sequence analysis revealed mixed culture			
FC4. 9	ON756101	*Photobacterium frigidiphilum* SL13 (AY538749)	99.47	91.8
FC4. 10	ON756102	*Photobacterium frigidiphilum* SL13 (AY538749)	99.63	92.7
FC4. 11	Sequence analysis revealed mixed culture			
FC4. 12	ON756103	*Halomonas titanicae* BH1 (AOPO01000038)	99.92	92.7
FC4. 13	ON756104	*Halomonas titanicae* BH1 (AOPO01000038)	100.00	91.8
FC4. 14	ON756105	*Sporosarcina aquimarina* SW28 (AF202056)	99.88	57.2
FC4. 15	Sequence analysis revealed mixed culture			
FC4. 16	ON773165	*Cobetia amphilecti* KMM 1561 (AB646236)	100.00	93.2
FC4. 17	ON773194	*Pseudomonas neustonica* SSM26 (KU716040)	99.78	94.9
FC4. 18	ON756108	*Shewanella piezotolerans* WP3 (CP000472)	98.68	93.1
FC4. 19	Sequence analysis revealed mixed culture			
FC4. 20	Sequence analysis revealed mixed culture			

Theses strains were phylogenetically identified by PCR amplification and sequences of the 16S ribosomal subunit. Sequencing data showed that thirteen of the strains were monocultures and that the remaining seven were still mixed cultures.

### Propane detection in strains isolated from marine sediment enrichment cultures

**Figure 2:**
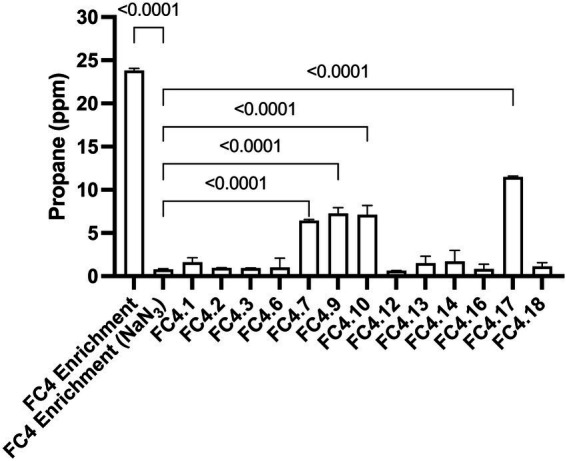
Propane production by 13 individual bacterial strains isolated from enrichment cultures generated from marine sediment samples. Following growth at 4°C for 8 weeks significantly higher levels of propane was detected in strains FC4.7, FC4.9, FC4.10 and FC4.17 in comparison to NaN_3_ treated control samples. One-way ANOVA with Dunnett’s *post hoc* test (*p* ≤ 0.05, *n* = 3).

Each of the 13 strains isolated was cultured in liquid media at 4°C for 8 weeks and analyzed for propane production by GC-FID. Four of the strains (FC4.7, FC4.9, FC4.10, and FC4.17) were shown to produce propane at a level significantly higher than the NaN_3_ negative controls (Figure 2). Interestingly none of these individual strains were able to produce the levels of propane observed in the original mixed microbial culture. FC4.7, FC4.9, and FC4.10 were all phylogenetically identified to be highly similar to the same strain of *Photobacterium frigidiphilum,* and FC4.17 was similar to *Pseudomonas neustonica* (Table 2). Repeated analysis for propane production showed those strains grouping to *Photobacterium* to be more consistent in growth and propane production than *Pseudomonas* sp. FC4.17, therefore *Photobacterium* sp. FC4.9 was selected for further characterization.

### Phylogeny of *Photobacterium* sp. FC4.9

**Figure 3:**
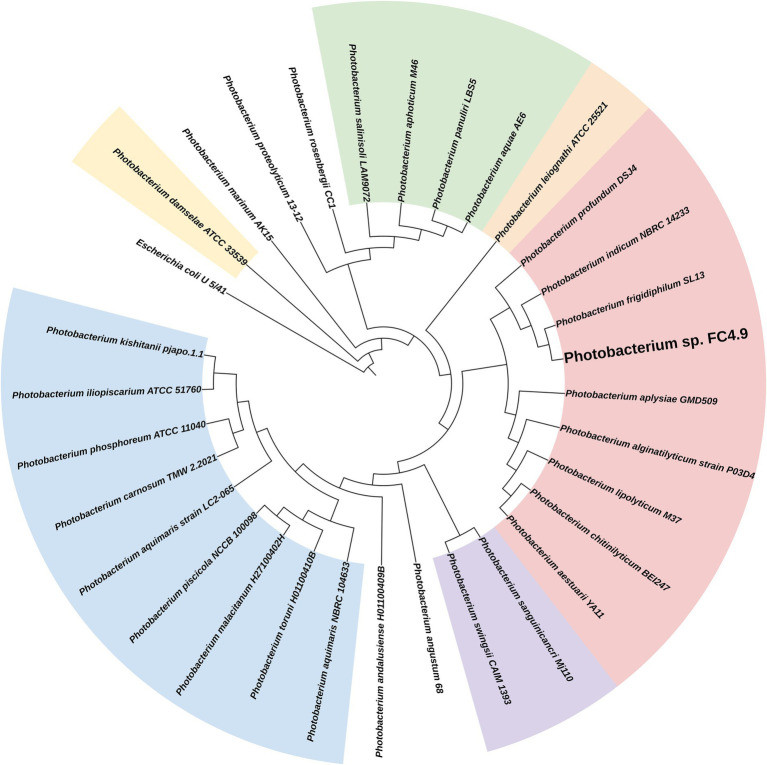
Phylogenetic tree generated from 16S rRNA gene sequence alignment. Within the *Photobacterium* genus, the propane producing strain *Photobacterium* sp. FC4.9 grouped to the Profundum clade. The phylogenetic tree was generated from an alignment of the 16S rRNA gene sequence from PCR amplified *Photobacterium* sp. FC4.9 DNA against the 16S rRNA gene sequences of the type strains of the 30 species within the genus.

**Figure 4:**
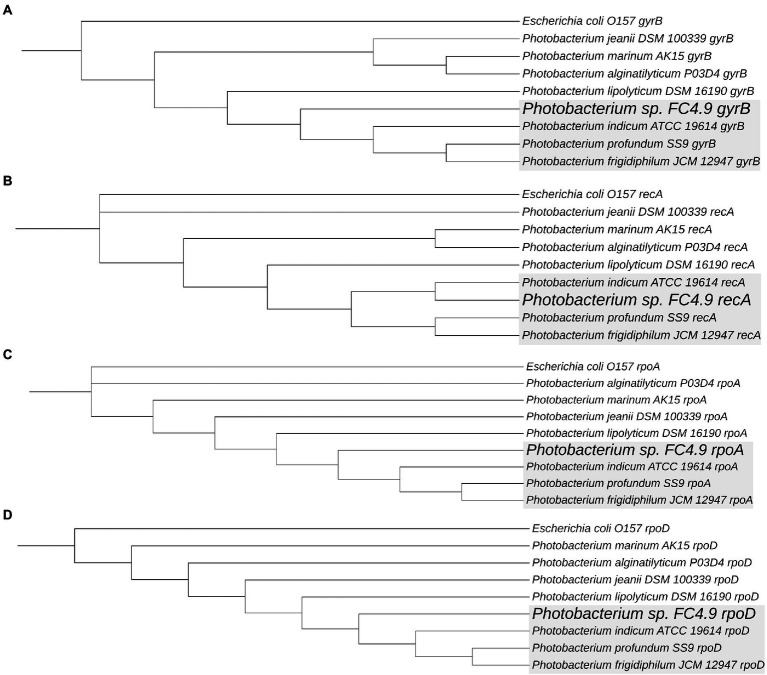
*Photobacterium sp.* FC4.9 was shown to be closely related to *P. indicum*, *P. profundum* and *P. frigidiphilum*. Phylogenetic trees were generated from multiple sequence alignments of *Photobacterium sp.* FC4.9 genes *gyrB* **(A)**, *recA* **(B)**, *rpoA* **(C)** and *rpoD* **(D)**, obtained from whole genome sequences, against the 7 Type Species within the *Profundum* clade of the genus. All trees were routed to respective gene sequences from *Escherichia coli* O157.

Sequencing of the 16S rRNA gene amplified from DNA extracted from FC4.9 showed this strain to belong to the genus *Photobacterium* (Table 2). Comparison *via* multiple sequence alignment to the 16S rRNA gene sequences from type strains of the recognized species within this genus showed *Photobacterium* sp. FC4.9 to group within the Profundum clade and be closely related to the species *Photobacterium profundum, Photobacterium indicum,* and *Photobacterium frigidiphilum* (Figure 3). To further investigate phylogeny and for future investigation of propane biosynthesis pathways, the genome of *Photobacterium* sp. FC4.9 was sequenced *via* Illumina sequencing. The commonly utilized phylogenetic reference genes *gyrB rpoD*, *recA*, and *rpoA* (Rocha et al., 2015) were identified within the whole genome sequence (WGS) and subsequently uploaded to the NCBI GeneBank database, (accession numbers ON803504, ON803505, ON803506, and ON803507, respectively). Multiple sequence comparisons using these reference genes to those of each member with the Profundum clade of the *Photobacterium* genus revealed *Photobacterium* sp. FC4.9 to have a close identity to *Photobacterium indicum* (Figure 4).

### Substrate usage for microbial propane production

**Figure 5:**
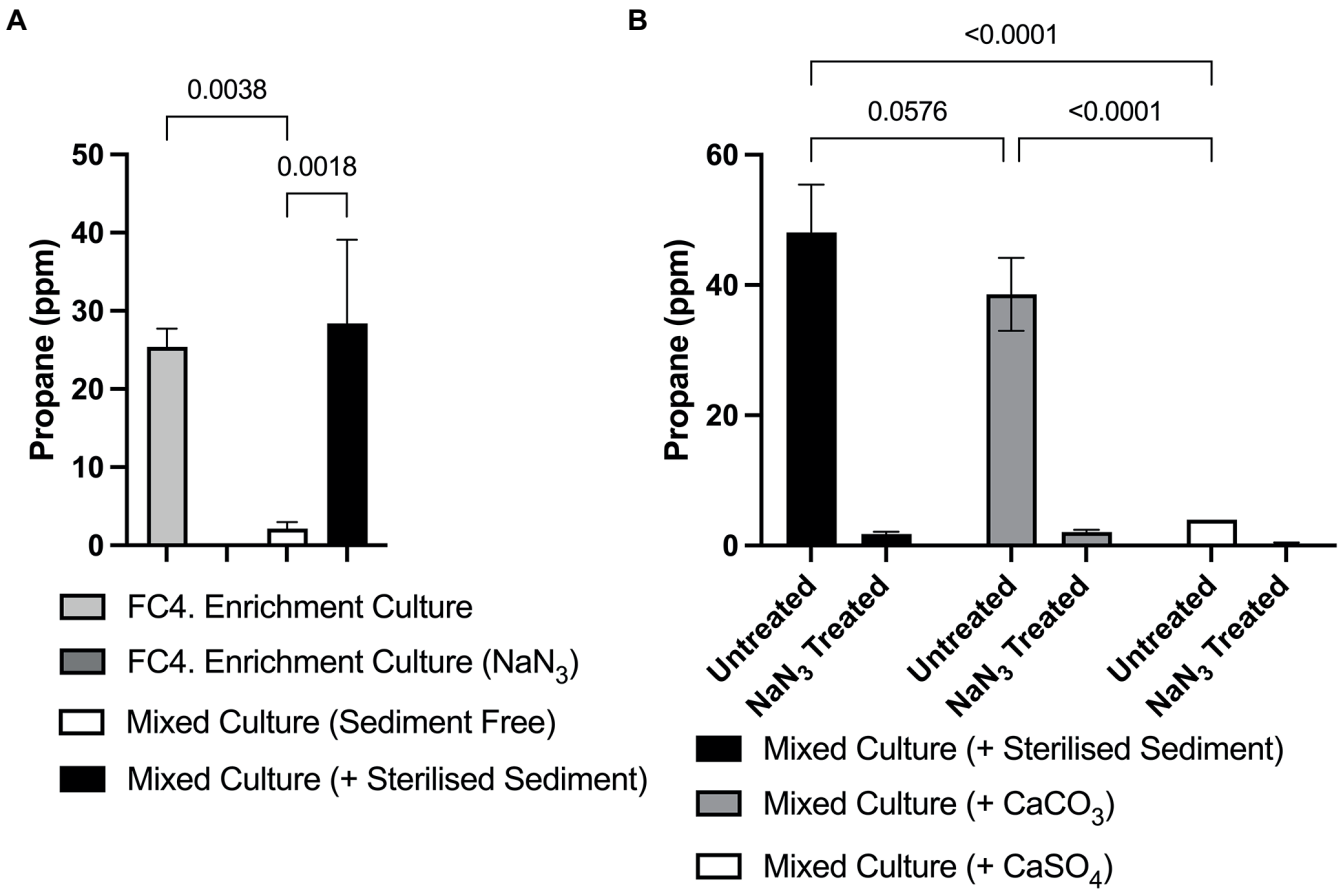
Propane production in marine sediment enrichment cultures requires either supplementation with sterilized marine sediment or CaCO_3_. **(A)** Significantly higher amounts of propane were detected both in initial cultures containing marine sediment, and in enrichment cultures supplemented with sterile marine sediment compared to an enrichment culture where sediment had been removed and not replaced by supplementation of the growth medium. **(B)** Significantly higher amounts of propane were detected in enrichment cultures supplemented with either sterile sediment or CaCO_3_ compared to either the NaN_3_ treated negative control samples or enrichment cultures supplemented with CaSO_4_. One-way ANOVA with Dunnett’s *post hoc* test (*p* ≤ 0.05, *n* = 3).

In early experiments, we observed that bacterial growth and/r propane production were not maintained over several rounds of sub-culture in fresh media. To determine whether the marine sediment itself, which was present in initial cultures, contained required nutrients for propane production, a sediment-free inoculum was prepared from initial cultures by gently resuspending microbial cells by gentle agitation. This was used to inoculate fresh media either devoid of any sediment or supplemented with pre-sterilized marine sediment (2% w/v). Significantly increased propane production was observed in cultures supplemented with pre-sterilized marine sediment compared to the cultures without sediment supplementation and to the NaN_3_ negative controls (*p* = 0.0038 and 0.0018, respectively, Figure 5A). Similar analysis showed that the same sterilized marine sediment was the requirement for propane production within monocultures of *Photobacterium* sp. FC4.9 (Data not shown).

Having observed that supplementation with pre-sterilized marine sediment was necessary for propane production, we hypothesized that the bacteria were utilizing carbonate or sulfate substrates present in the sediment in propane biosynthesis. Therefore, we investigated propane production in cultures supplemented with either 0.02 M CaCO_3_ or 0.02 M CaSO_4_. We found that significant amounts of propane were produced in cultures supplemented with CaCO_3_ (38.53 ppm ± 5.61) compared to the NaN_3_ negative controls (2.03 ppm ± 0.34), with amounts statistically comparable to cultures supplemented with pre-sterilized marine sediment (48.05 ppm ± 7.38). In contrast, significantly less (3.97 ppm ± 0.007) propane was produced in cultures supplemented with CaSO_4_ compared to cultures supplemented with either pre-sterilized sediment or CaCO_3_. These results indicate that inorganic carbonate was required for propane production (Figure 5B). As before similar results were obtained when using monocultures of *Photobacterium* sp. FC4.9.

## Discussion

This study identified both mixed microbial populations isolated from a Western English Channel marine sediment sample and a single bacterial strain from the same sample that could produce propane. The successful isolation of both mixed microbial cultures and individual bacterial strains from a multitude of environmental niches where propane production is predicted to occur shows that these environments possess rich microbial populations. It is likely however that the mixed microbial cultures and strains isolated by this study only represent a fraction of the microbial flora present within these environments as many bacterial and archaea cannot be routinely grown under laboratory conditions. As such one future avenue of this research will be to fully characterize the microbial population present within this marine sediment sample *via* a meta-genomic approach utilizing third-generation DNA sequencing. With regards to the lack of growth in the Canadian gas field sample we predict this is an artifact from the transport of the sample.

Propane production was identified in live microbial cultures, analyzing the headspace above the culture by GC–FID. FID is a sensitive detection method and has advantages over, e.g., gas chromatography–mass spectrometry, where without accurate mass measurement it is not possible to distinguish propane at low concentrations from carbon dioxide and other background ions (Fancy et al., 2006; Ligor and Buszewski, 2008). In order to be confident that the compound we were detecting in our microbial cultures was indeed propane, a validated propane standard was utilized and analysis was repeated in a different GC column possessing different column chemistry (GS GasPro SiPLOT column). In all cases, propane was not detected in negative control samples or samples inoculated with live cells and treated with a bactericide, therefore indicating that the propane is microbially biosynthesized. The ability of microbial propane production in the marine environment is not unprecedented. For example, concentrations and isotopic compositions of ethane and propane in deep sediments from the south-eastern pacific are best explained by microbial production of these gasses *in situ*, with a feasible mechanism involving the reduction of acetate to ethane (Hinrichs et al., 2006). Propane is enriched in ^13^C relative to ethane, the amount being consistent with derivation of the third C from inorganic carbon dissolved in sedimentary pore waters, with reactions yielding free energy sufficient for growth (Hinrichs et al., 2006). A second plausible mechanism with propane formed in a reaction analogous to methane formation, where acetate and carbonate may act as a precursor of propane in the same way that carbon dioxide is a precursor of methane (Hinrichs et al., 2006). Methanogenesis in Archaea is carried out by the enzyme Methyl Co-enzyme M Reductase (MCR). Its substrate is methane thiol, but C2-C4 thiols also fit the active site of MCR (Oremland, 1981; Oremland et al., 1988).

The discovery of a naturally occurring propane-producing bacterium offers scope for culture optimization and scale-up toward a fermentation route for bio-propane production. The observation that propane production was higher in a mixed microbial population compared to a monoculture of *Photobacterium* sp. FC4.9 alone may indicate evidence of syntropy, with a metabolite synthesized by one species in the mixed culture being utilized as raw material by another. However, in general, the propane yields in both mixed and mono-species cultures were low (~ 23 ppm for the mixed species). The calculated mg propane per liter fermentation was 0.044 mg L^−1^, a 4,545 fold lower yield than that obtained through synthetic biology routes where maximum published propane yields are *ca.* 0.2 g L-1 fermentation (Menon et al., 2015). However, microbial growth was very likely limited by nutrient limitation in our un-optimized culture conditions at only a 10 ml scale. *In situ* environmental conditions are likely to differ greatly from those *in vivo* and may be more favorable for propane production, resulting in greater quantities produced. Due to the low yield of production, it is considered more likely that elucidation of the biosynthetic pathway and identification of relevant genes and enzymes utilized by this strain will contribute to the synthetic biology toolbox for genetically engineered pathways within a host strain that can grow and produce propane under more favorable conditions.

To elucidate potential propane biosynthesis pathways a subsystem analysis of the WGS of *Photobacterium* sp. FC4.9 was carried out using the Comprehensive Genome Analysis Service provided by Pathosystems Resource Integration Center (PATRIC) (Brettin et al., 2015). An interesting finding was that propane production by *Photobacterium* sp. FC4.9 only occurred when media was supplemented with sterilized marine sediment derived from the original site of isolation (the Western English Channel) or CaCO_3_. Our functional analysis identified genes based on the specific biological process hypothesized to be putative orthologs for two carbonate transporters, *bicA*_1 and *bicA*_2 (ON859037, ON859038); and a putative ortholog of carbonic anhydrase, *cah* (ON859039) (Supuran and Capasso, 2017; Wang et al., 2019). Enzymes expressed from these genes are known to be functional in Cyanobacteria where CO_2_ is converted to 3-carbon molecules and oxygen using the RuBisCO enzyme (Espie and Kimber, 2011). They are involved in actively transporting carbonate to carboxysomes in the cell and converting it to CO_2_, providing a high concentration of CO_2_ at the location of the enzyme. This is important in terms of efficiency because RuBisCO also catalyzes the reverse reaction and has a higher affinity for O_2_ than CO_2_. Carboxysomes are icosahedral compartments in the cell and also occur in many autotrophic bacteria including *Gammaproteobacteria* such as *Photobacteria* (Espie and Kimber, 2011; Scott et al., 2020). The carbonate transport-carbonic anhydrase system is of interest in Synthetic Biology and incorporation of this module to increase CO_2_ assimilation to overcome some limitations in fermentation technology has been recently described (Flamholz et al., 2020; Franklin and Jonikas, 2020; Zhang et al., 2021). The same subsystem functional analysis of *Photobacterium* sp. FC4.9 WGS data also revealed orthologs of the genes *selB* (ON859041), a Selenocysteine-specific elongation factor; *selA* (ON859040), an L-seryl-tRNA(Sec) selenium transferase; selD_1 and selD2 (ON859042, ON859043), Selenide water dikinases; and selU (ON859044), a tRNA 2-Selenouridine synthase (Forchhammer and Bock, 1991; Veres et al., 1992; Leibundgut et al., 2005; Böck et al., 2006; Sierant et al., 2018). The enzymes and elongation factors expressed by these genes are involved in mechanisms for Selenocysteine (Sec) production (where Se replaces the S in Cysteine): Selenocysteine has a lower reduction potential than Cysteine so it is possible that the reaction thermodynamics for propane production is more favorable if Selenocysteine enzymes are involved (Sun et al., 2013; Fu et al., 2018). This evidence suggests that incorporation of Se compounds into the culture media may further optimize propane production by *Photobacterium* sp. FC4.9 and as such this is the subject of our ongoing research into this bacterium.

To conclude, this study has identified, for the first time, a naturally occurring, propane-producing bacterium, *Photobacterium* sp. FC4.9. The study also identified several genes within the genome of this strain that are potentially involved in the propane biosynthesis pathway. As such this discovery opens the possibility of an alternative production pathway, *via* microbial fermentation, for the generation of propane for BioLPG. However, the quantity of propane produced was limited, and while there is scope for optimization and scale-up toward a fermentation route for bio-propane production, it is considered more likely that further elucidation of the mechanisms of propane biosynthesis by *Photobacterium* sp. FC4.9 could be utilized to generate recombinant bacteria with optimal production yields.

## Data availability statement

The data presented in the study are deposited in the NCBI Nucleotide repository, accession numbers are provided in the main text of the article.

## Author contributions

FC: methodology, investigation, and writing – original draft. MT: formal analysis, writing – original draft, and writing – review and editing. NH: investigation. KS: funding acquisition, conceptualization, resources, and writing –review and editing. RM: conceptualization, supervision, and writing – review and editing. IB: project administration, funding acquisition, conceptualization, supervision, and writing – review and editing. All authors contributed to the article and approved the submitted version.

## Funding

This research was jointly funded through an industrial partnership with SHV Energy (Capellalaan 65, Hoofddorp, 2132 Jl, The Netherlands); Invest Northern Ireland Proof of Concept grant 909 (Bio-propane); and a Department for the Economy (NI)/SHV Energy Co-operative Awards in Science and Technology (CAST) Ph.D. studentship.

## Conflict of interest

The authors declare that this study received funding from SHV Energy. The funder was not involved in the study design, collection, analysis, interpretation of data, the writing of this article, but were aware of the findings and encouraged us to submit for publication.

KS was employed by the company SHV Energy.

The remaining authors declare that the research was conducted in the absence of any commercial or financial relationships that could be construed as a potential conflict of interest.

## Publisher’s note

All claims expressed in this article are solely those of the authors and do not necessarily represent those of their affiliated organizations, or those of the publisher, the editors and the reviewers. Any product that may be evaluated in this article, or claim that may be made by its manufacturer, is not guaranteed or endorsed by the publisher.
