# Supplementary material for: Biogenic propane production by a marine Photobacterium strain isolated from the Western English Channel
Source: Front Microbiol. 2022 Oct 25;13:1000247. doi: 10.3389/fmicb.2022.1000247 (PMC9642325; doi:10.3389/fmicb.2022.1000247)
Supplement: Supplementary file 1 [file Data_Sheet_1.DOCX]

Supplementary Material

#
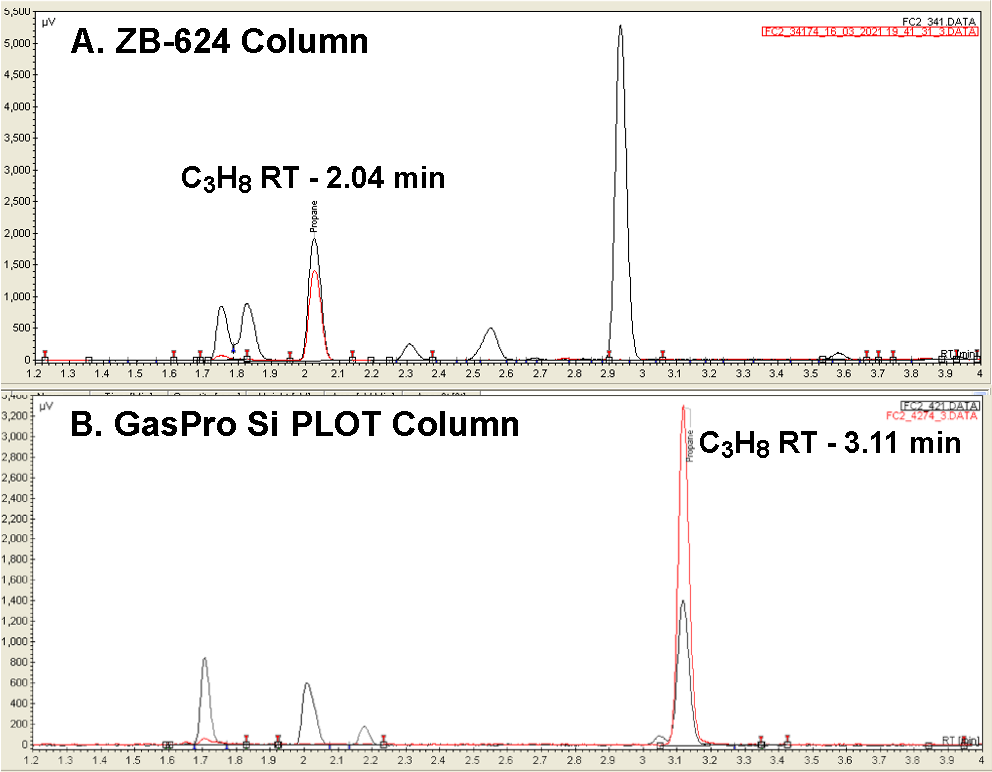


**Supplementary Figure 1.** GC-FID chromatograms showing the retention time (RT) of propane detected in enrichment cultures generated from marine sediment samples (Black trace) and a commercially available propane standard (*Merck*) (Red trace). When run GC columns with two difference column chemistries (A) ZB-624 (*Phenomenex*) and (B) GasPro Si PLOT (*Agilent*) both standard and samples possessed the same RT.

**Supplementary Table 1.** Media components and protocol for the generation of modified R2A Medium utilized in this study.

| Modified Marine Medium Base | | |
| --- | --- | --- |
| Component | **Amount (g L^-1^)** | **Supplier** |
| Protease Peptone | 1.5 | *Merck* |
| Casamino acids | 1.5 | *Merck* |
| Yeast Extract | 1.5 | *Merck* |
| Glucose | 1.5 | *Merck* |
| Soluble Starch | 1.5 | *Merck* |
| Na-pyruvate | 0.3 | *Merck* |
| MgSO_4_. 7H_2_O | 0.05 | *Merck* |
| K_2_HPO_4_ | 0.9 | *Merck* |
| Tween 80 | 0.05% (v/v) | *Merck* |

**Supplementary Table 2.** Media components and protocol for the generation of modified Zobel Marine Medium utilized in this study.

| Modified Marine Medium Base | | |
| --- | --- | --- |
| Component | **Amount (g L^-1^)** | **Supplier** |
| Protease Peptone | 0.5 | *Merck* |
| Casamino acids | 0.5 | *Merck* |
| Yeast Extract | 0.5 | *Merck* |
| NaCl | 26.29 | *Merck* |
| KCl | 0.74 | *Merck* |
| CaCl_2_ | 0.99 | *Merck* |
| MgCl_2_. 6H_2_O | 6.09 | *Merck* |
| MgSO_4_. 7H_2_O | 3.93 | *Merck* |
| K_2_HPO_4_ | 0.2 | *Merck* |
| Sterilise via autoclave at 121°C for 15 min and add 10 mL 100 X Marine Supplements | | |
|  | | |
| 100 X Marine Supplements | | |
| Component | **Amount (% v/v)** | **Supplier** |
| Trace Element Stock Solution | 1 | N/A |
| Vitamin Stock Solution | 1.25 | N/A |
| Nicotinamide | 1 mM | *Merck* |
|  | | |
| Trace Element Stock Solution | | |
| Component | **Amount (g L^-1^)** | **Supplier** |
| Na_2_EDTA | 43 | *Merck* |
| FeCl_3_. 6H_2_O | 31.5 | *Merck* |
| ZnSO_4_. 7H_2_O | 0.22 | *Merck* |
| CoCl_3_. 6H_2_O | 0.1 | *Merck* |
| MnCl_2_. 4H_2_O | 1.8 | *Merck* |
| NaMoO_4_. 2H_2_O | 6.3 | *Merck* |
| Sterilise through 0.2 μm filter | | |
|  | | |
| Vitamin Stock Solution | | |
| Component | **Amount (g L^-1^)** | **Supplier** |
| B12 cyanocobalamin | 0.005 | *Thermo-Scientific* |
| B1 Thiamine HCl | 1 | *Merck* |
| Biotin | 0.05 | *Merck* |
| Sterilise through 0.2 μm filter | | |

**Supplementary Table 3.** Gas chromatography conditions for propane analysis

| **Injector Temperature** | 250 °C | | |
| --- | --- | --- | --- |
| **Detector Temperature** | 250 °C | **Combustion Gas Composition** | H 30 mL min^-1^  air 300 mL min^-1^;  make up gas: 29 mL min^-1^ |
| **Carrier Gas** | Helium | **Carrier Gas Flow Rate** | 1.2 mL min^-1^ |
|  |  | **Carrier Gas Linear Flow** | 39 cm s^-1^ |
| **Injection volume** | 250 µL | **Split ratio** | 20 |
| **Pressure Pulse** | 68.95 kPa | **Pulse duration** | 0.25 min |
| **Sample agitation prior to injection** | 30 s, 30 °C | | |
| **Temperature gradient**  **ZB-624 column** | \| Rate  (°C min^-1^) \| Temperature  (°C) \| Hold  (min) \| Total time  (min) \| \| --- \| --- \| --- \| --- \| \| Initial \| 30 \| 2 \| 2 \| \| 20 \| 120 \| 2 \| 8.5 \| \| 20 \| 30 \| 0 \| 13 \| | | |
| **Temperature gradient**  **GasPro SI PLOT column** | \| Rate  (°C min^-1^) \| Temperature  (°C) \| Hold  (min) \| Total time  (min) \| \| --- \| --- \| --- \| --- \| \| Initial \| 90 \| 4 \| 4 \| \| 20 \| 150 \| 2.5 \| 9.5 \| \| 20 \| 90 \| 2 \| 14.5 \| | | |
