# Supplementary figures and images for: Biogenic propane production by a marine Photobacterium strain isolated from the Western English Channel
Source: Front Microbiol. 2022 Oct 25;13:1000247. doi: 10.3389/fmicb.2022.1000247 (PMC9642325; doi:10.3389/fmicb.2022.1000247)

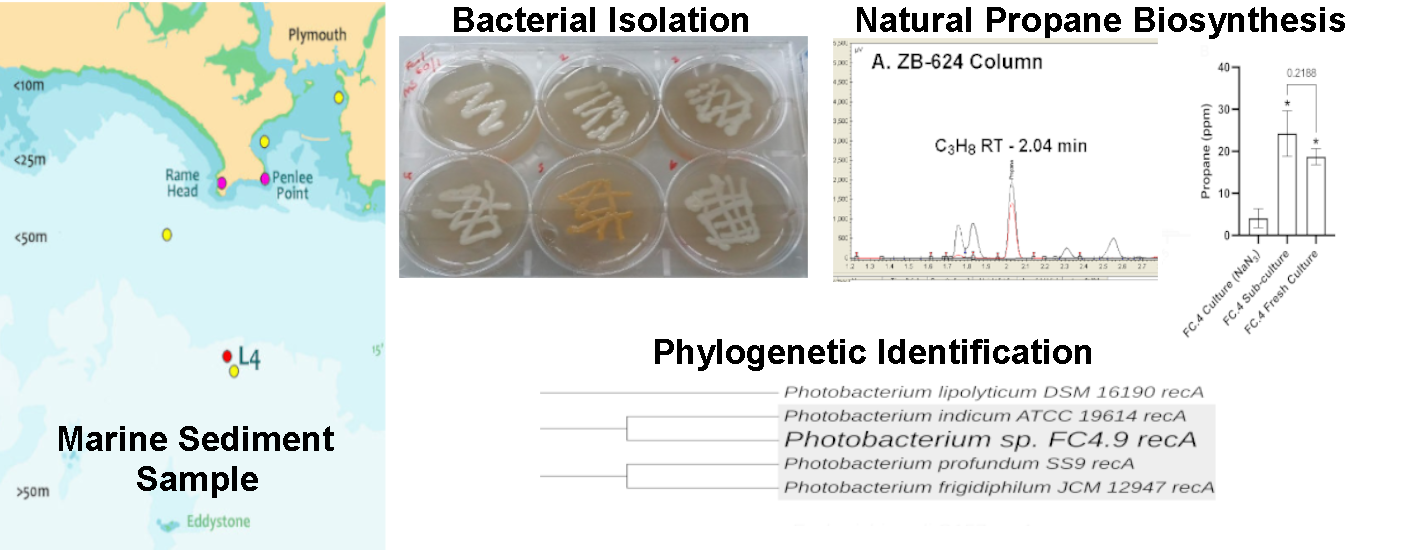

Supplement: Supplementary file 2 [file Image_1.TIF]
